# Subcontinental heat wave triggers terrestrial and marine, multi-taxa responses

**DOI:** 10.1038/s41598-018-31236-5

**Published:** 2018-08-30

**Authors:** Katinka X. Ruthrof, David D. Breshears, Joseph B. Fontaine, Ray H. Froend, George Matusick, Jatin Kala, Ben P. Miller, Patrick J. Mitchell, Shaun K. Wilson, Mike van Keulen, Neal J. Enright, Darin J. Law, Thomas Wernberg, Giles E. St. J. Hardy

**Affiliations:** 1 Centre of Excellence for Climate Change, Woodland and Forest Health, School of Veterinary and Life Sciences, Murdoch University, Perth, Western Australia Australia; 2 Kings Park Science, Department of Biodiversity, Conservation and Attractions, 1 Kattidj Close, Kings Park, Western Australia Australia; 3 School of Natural Resources and the Environment, University of Arizona, Tucson, Arizona United States of America; 4 Department of Ecology and Evolutionary Biology via joint appointment, University of Arizona, Tucson, Arizona United States of America; 5 Environmental and Conservation Sciences, School of Veterinary and Life Sciences, Murdoch University, Perth, Western Australia Australia; 6 Centre for Ecosystem Management, Edith Cowan University, Joondalup, Western Australia Australia; 7 School of Plant Biology, University of Western Australia, Crawley, Western Australia Australia; 8 CSIRO Land and Water, Sandy Bay, Tasmania Australia; 9 Department of Biodiversity, Conservation and Attractions, Kensington, Perth, Western Australia Australia; 10 Oceans Institute, University of Western Australia, Crawley, Western Australia Australia

## Abstract

Heat waves have profoundly impacted biota globally over the past decade, especially where their ecological impacts are rapid, diverse, and broad-scale. Although usually considered in isolation for either terrestrial or marine ecosystems, heat waves can straddle ecosystems of both types at subcontinental scales, potentially impacting larger areas and taxonomic breadth than previously envisioned. Using climatic and multi-species demographic data collected in Western Australia, we show that a massive heat wave event straddling terrestrial and maritime ecosystems triggered abrupt, synchronous, and multi-trophic ecological disruptions, including mortality, demographic shifts and altered species distributions. Tree die-off and coral bleaching occurred concurrently in response to the heat wave, and were accompanied by terrestrial plant mortality, seagrass and kelp loss, population crash of an endangered terrestrial bird species, plummeting breeding success in marine penguins, and outbreaks of terrestrial wood-boring insects. These multiple taxa and trophic-level impacts spanned >300,000 km^2^—comparable to the size of California—encompassing one terrestrial Global Biodiversity Hotspot and two marine World Heritage Areas. The subcontinental multi-taxa context documented here reveals that terrestrial and marine biotic responses to heat waves do not occur in isolation, implying that the extent of ecological vulnerability to projected increases in heat waves is underestimated.

## Introduction

Recent dramatic ecological shifts in response to climate extremes have had profound societal impacts^1^ and have galvanized recognition of extreme climate events, rather than gradual, mean change, as the most conspicuous hand of climate change^1,2^. Of particular concern are short and extreme temperature anomalies spanning days to weeks, referred to collectively here as “heat waves”^3^. A terrestrial heat wave event can be identified as three or more consecutive days where the maximum temperature is over the 90^th^ percentile^4^, whereas a marine heat wave is usually defined as a discrete, prolonged, anomalously warm water event in a particular location^5^. The land area affected by heat waves is expected to double by 2020 and quadruple by 2040^6^. In combination with droughts, heat waves are also predicted to increase in frequency and magnitude, with climate models pointing at land–atmosphere coupling as a key reason for this exacerbation^7,8^. Increases in frequency are not restricted to terrestrial heat waves; marine heat waves now occur 4–5 times more often than in the 1980s^9^.

Heat waves can produce profound physiological consequences for flora and fauna^2,10,11^, triggering mortality, abrupt demographic and community-level disruptions, and ecosystem reconfigurations^12–14^ – as also reflected in well-documented spikes in human mortality during recent heat waves^1,12^. Despite the potential subcontinental scale of heat waves^15^, studies of heat wave effects on biota almost exclusively focus on physiological or phenological change to a single species or ecosystem type^10,16–18^. This precludes broader evaluation of their impacts, which could disrupt biota across taxonomic and evolutionary lineages, multiple trophic levels, and fundamentally different ecosystem types. In terrestrial ecosystems, research has focused on the combined effects of drought and heat on tree die-off^17^, with more recent studies pointing to the specific importance of heat waves^16^. However in marine ecosystems, heat waves alone are recognised as obvious and dramatic drivers of extensive coral bleaching and mortality^19–21^. Long-term, gradual climate-driven changes in growth chronology have been reported within individuals from both terrestrial and marine organism systems^22^. But, despite the potential for heat waves to straddle terrestrial and marine ecosystems concurrently^15,23^ their ability to simultaneously trigger ecological responses in both ecosystems at the ecoregion and sub-continental scale has received limited attention. Furthermore, although heat waves are explicitly discussed in terms of impacts on human populations in the most recent climate assessment reports, their full and detailed effects on ecosystems is lacking^1,12^. Given these gaps in reporting and knowledge, the full extent of ecological vulnerability to projected heat waves may be underestimated.

Following a heat wave event in early 2011, which straddled both the marine and terrestrial ecosystems of Western Australia, we aimed to document, using a meta-analytic framework, the pervasive ecological effect of a climate change-induced extreme event, highlight the breadth of taxa affected, and quantify demographic change in the abundance and mortality rates. We predicted that the heat wave could cause the loss of foundation species, demographic shifts, as well as alter the composition, structure and function of ecosystems, and change species distributions across a wide range of taxa. That is, overall, some taxa would respond positively, others negatively. Organisms present prior to the event (sessile species, long-lived vagile taxa) were assumed *a priori* to be negatively impacted, whereas vagile consumers that were not present or rare pre-heat wave were presumed to be neutral or increasing following a heat wave event.

## Results

### Climatic event

**Figure 1:**
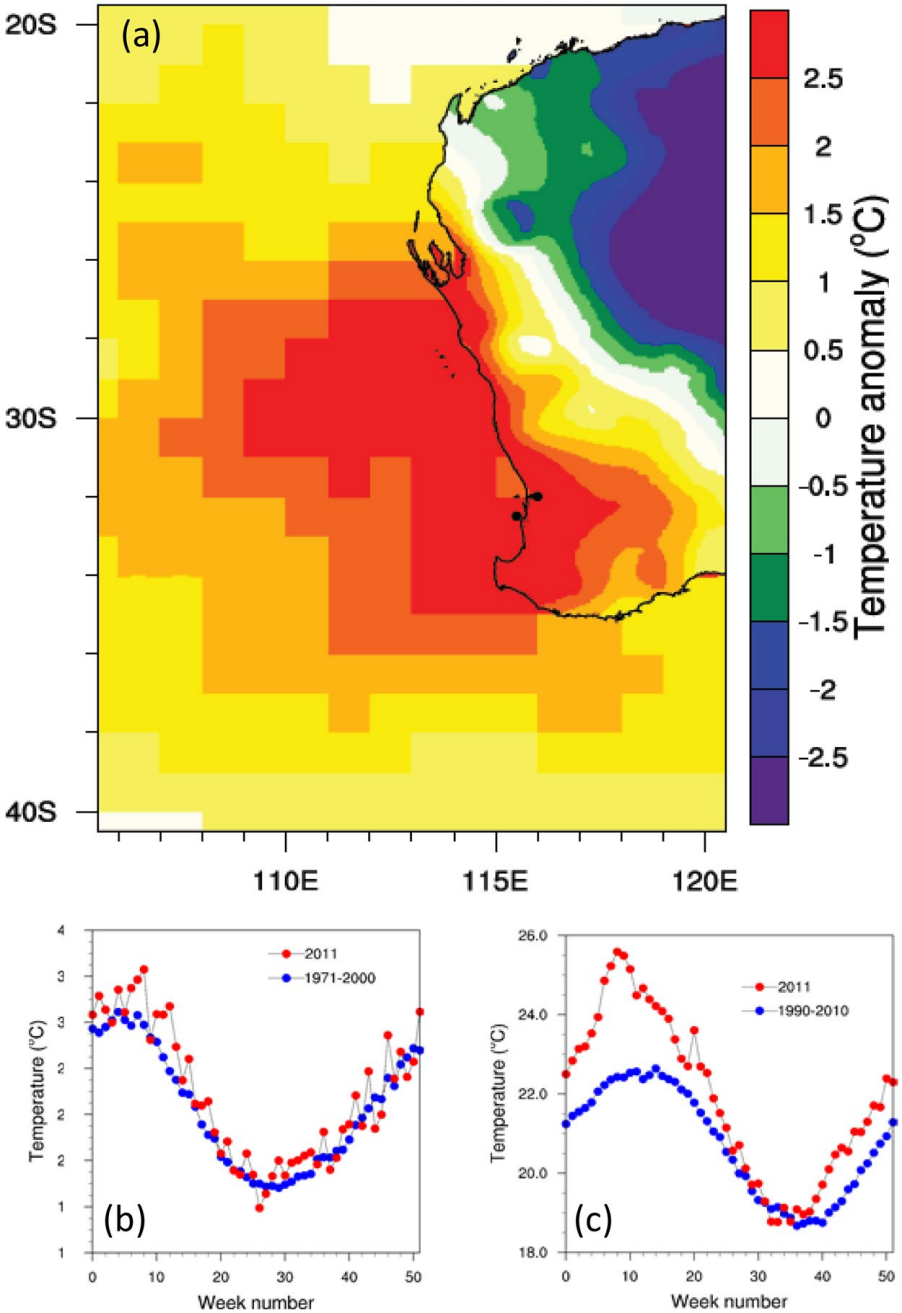
Temperature anomalies for Western Australia in early 2011. (**a**) Maximum temperature anomaly over land from gridded observations^63^ for March 2011 relative to March 1971–2000. Sea Surface Temperature (SST) anomaly from a combined *in-situ* and satellite derived product^67^ for March 2011 relative to 1971–2000. (**b**), Weekly mean maximum temperatures during 2011 (red dots), and the mean over 1971–2000 (blue dots) near Perth (32 °S, 116 °E) as shown by the black dot in panel (a,c) Weekly mean SST during 2011 and the mean over 1990–2010 off the coast of Western Australia (−32.5 °S, 115.5 °E). Figure was created with the NCAR Command Language (Version 6.4.0) [Software]. (2017). Boulder, Colorado: UCAR/NCAR/CISL/TDD. http://dx.doi.org/10.5065/D6WD3XH5.

 The terrestrial maximum temperatures extending from 25°S at Shark Bay to 34°S at Cape Leeuwin for all of March 2011, were 2 °C higher than the long-term March average over the period 1971–2000 baseline (Fig. 1a). Minimum temperatures were also higher than average (see Fig. S1). At finer temporal and spatial scales, weekly maximum temperature near the Western Australian city of Perth (32°S, 116°E) exceeded long-term means by ~5 °C (Fig. 1b). The number of heat wave days in 2011 was the highest, and the Standardized Precipitation Evapotranspiration Index (SPEI)^24^ was the lowest on record since 1960 (Fig. S2a,b). The terrestrial heat wave coincided with a drought characterized by an extremely dry 2010 winter (40–50% below the average rainfall^25^) and a 30-year pronounced drying trend of reduced winter rainfall (14% decline)^26^.

The conditions of the terrestrial heat wave were mirrored in marine conditions, and coincided with a near-record strength Leeuwin Current and one of the strongest La Niña events on record^3^. Sea surface temperatures (SST) were abnormally high^27^ and also nested within a long-term increase in mean SST for Western Australia^28^. The SSTs for March 2011 were 2–2.5 °C higher compared with the long-term March average over the period 1971–2011 (Fig. 1a) and close to the coast, weekly temperatures were 3–3.5 °C above long term averages (1990–2010) for that time of year (Fig. 1c).

### Biotic response

**Figure 2:**
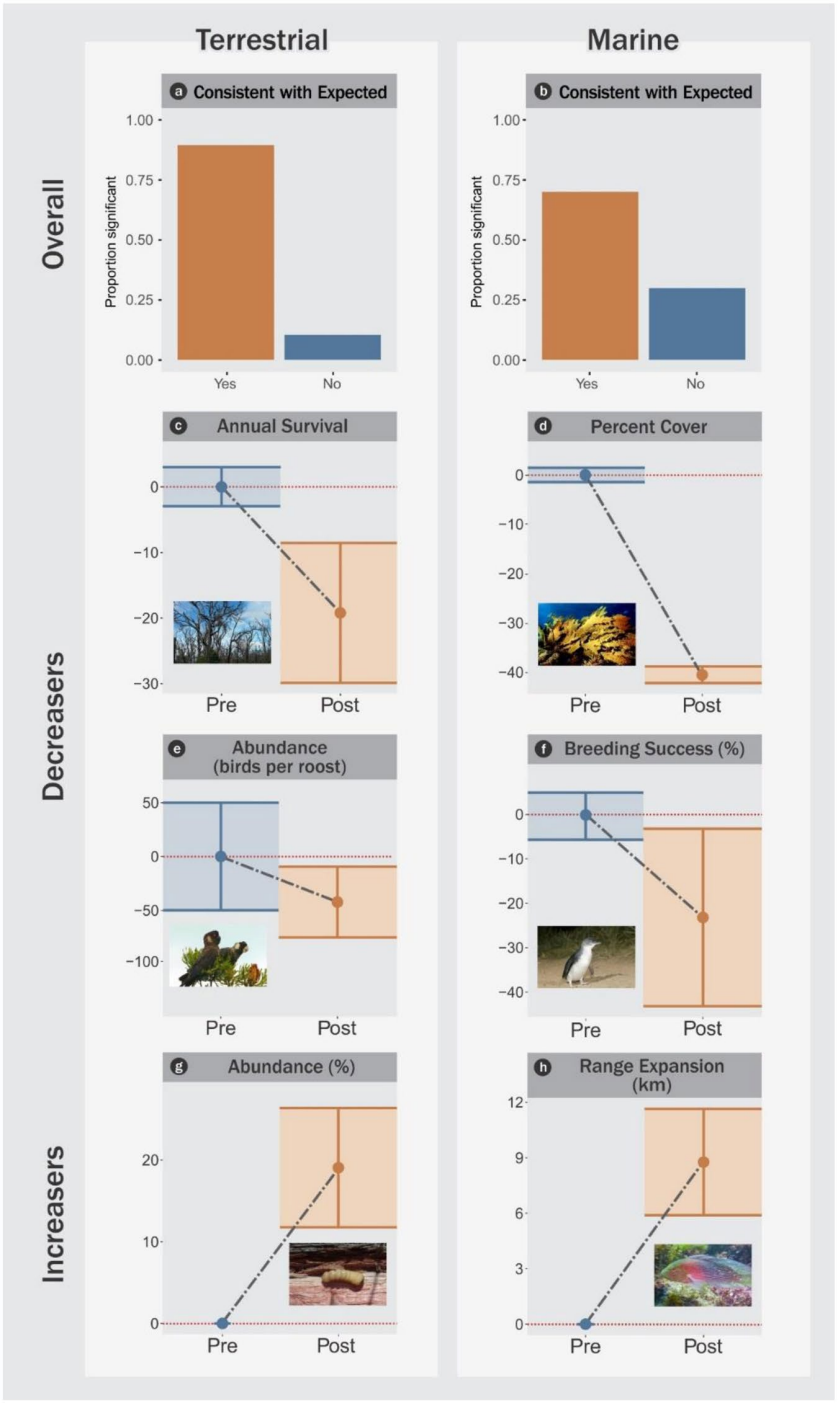
Heat wave-impacted organisms in terrestrial and marine ecosystems in Western Australia, 2011. (**a**,**b**) consistent with expected, (**c**–**f**) examples from species that decreased, and (**g**,**h**) increased. Blue dots and confidence interval (CI) lines denote prior to heat wave, orange dots and CI lines denote following the heat wave. Note that these are different types of responses (e.g. cover change, mortality) and are not connected in a cascade or food web, but are separate examples. Inset photos are an example of each type: (**c**) 12 species of trees plus 3 shrubs (photo credit: K. Ruthrof), (**d**) 3 species of seagrass/kelp (T. Wernberg), (**e**) 1 species of cockatoo (L. Valentine), (**f**) 1 species of penguin (B. Cannell), (**g**) 1 species of wood boring insect (G. Matusick) and, (**h**) 3 species of fish (T. Wernberg).

The subcontinental heat wave event triggered statistically significant, abrupt, and synchronous (i.e. occurring within 1–2 seasons) biotic disruptions in both terrestrial and marine ecosystems, including mortality, demographic shifts and altered species distributions (Fig. 2a,b). Of 19 terrestrial quantitative contrasts, 17 (89%) were significant and all were in the expected direction (Fig. 2a). Of 20 marine quantitative contrasts, 14 (70%) were significant and all but one were in the hypothesised direction (Fig. 2b).

When further refined into trophic levels, primary producers in both terrestrial (Fig. 2c) and marine (Fig. 2d) ecosystems showed decreases in both survival and cover, in response to the heat wave. Consumers were subdivided *a priori* into groups to differentiate those expected to decrease in abundance following a heat wave (sessile organisms, long-lived vertebrates) from those expected to increase in abundance or distribution (e.g. tropical fish). Consumers expected to be negatively affected by the heat wave did so for both the terrestrial (Fig. 2e) and marine (Fig. 2f) ecosystems (abundance of the endangered cockatoo *Calyptorhynchus latirostris*, and breeding success of penguins *Eudyptula minor* declined). Similarly, consumers expected to increase in abundance did so for both the terrestrial (Fig. 2g) and marine (Fig. 2h) ecosystems (outbreaks of the wood boring insect *Phoracantha semipunctata* in response to dying trees) reinforcing support for the pervasive effect of the heat wave in both biomes. Further underscoring the scale of the event, taxa from a breadth of lineages were impacted by the heat wave (Table 1; species-specific responses by study are provided in Extended Data Table 1), providing additional evidence for the permeating effects of the heat wave.

**Table 1:** Taxonomic and spatial sample of heat wave induced impacts and their duration and magnitude.

Domain	Trophic Level	Response group	Lifeform	N taxa	N quantitative studies	Latitude	Duration (seasons)	Response Magnitude	Units
Terrestrial	Producer	Decreasing	Understory shrubs	3	3	29.6S	2	−2.4	Survival
			Tree-emergent	2	2	29.6S	2	−17.6	Survival
			Tree-woodland	7	7	31.4–32.4S	2	−25.1	Survival
			Tree-forest	3	3	32.0–32.7S	2	−48.6	Survival
	Consumer	Decreasing	Bird	1	1	31.0–33.0S	1	−43.4	Abundance
		Increasing	Insect	1	1	32.0–32.7S	1	19.1	Abundance
Marine	Producer	Decreasing	Coral	3	3	20.5–32.0S	2	−10.9	Cover
			Macroalgae	4	3	30.3S	1	−21.4	Cover
			Seagrass	1	1	26.2S	1	−49.7	Cover
	Consumer	Decreasing	Shellfish	4	0	26.2–32.0S	1	NA	
			Bird	1	1	32.0S	2	−23.2	Breeding success
		Increasing	Fish	5	3	30.3–32.0S	1	6.6	Range extension

Note: terrestrial and marine ecosystems were impacted in Western Australia from January 2011 (see Extended Data Table 1 for individual studies).

The magnitude of changes documented was substantial. Mean shrub and tree mortality (including *Beaufortia elegans*, *Eucalyptus marginata* and *Corymbia calophylla*) was 19% following the heat wave (Fig. 2a), greatly exceeding the magnitude of background mortality (e.g. for *Eucalyptus marginata*, reported locally: 0.02–2.42%/yr^29^) and for forests elsewhere^30^. Annual counts of endangered Carnaby’s Black Cockatoo (*Calyptorhynchus latirostris)*, have to date, not recovered from the substantial decline (60%) observed (Fig. 2; Extended Data Table 1). Our quantitative results are reinforced by other qualitative studies indicating sub-lethal heat wave impacts, including partial tree canopy dieback^31^, coral bleaching^32^, decline in health status of turtles (*Chelonia mydas*)^18^, and altered lobster (*Panulirus cygnus*) behaviour^33^.

## Discussion

The results of our meta-analytic approach illustrate a broad range of diverse and pervasive biotic disruptions caused by a heat wave, reflecting strong spatial coherence between climate signal and ecological response. These include loss of foundation, habitat-forming species such as trees (e.g. *Eucalyptus marginata* and *Corymbia calophylla*^16^), corals (Acropora^34^), algae^35^, critical reductions in ionic vertebrate species such as an endangered cockatoo (*Calyptorhynchus latirostris*^36^), and penguins (*Eudyptula minor*^37^), increased abundance of fishes (e.g. *Labracinus lineatus*^35^), and an outbreak of wood boring insects (*Phoracantha semipunctata*^38^). Specifically, negative impacts of the heat wave were anticipated for taxa where individuals were physically present during the heat wave. For example, all sessile organisms, as well as longer-lived vertebrates such as Little Penguins (*Eudyptula minor*) and Carnaby’s Black Cockatoos (*Calyptorhynchus latirostris*). In contrast, a number of species increased dramatically from low numbers (tropical fish and wood boring beetles). The attribute of a species population being present prior to the heat wave provided a clear and useful way in which to consider heat wave response syndromes^39^. Qualitative studies describing responses to the heat wave also reinforced our quantitative results (Extended Data Table 1). Ecological changes triggered by heat waves like those we document here are likely to cause fundamental disruptions in the structure^40^, function^41^, and distribution of species leading to phase shifts^42^ or cascades^43^ to alternate ecological states with long-term consequences for ecosystem services^44^. For example, following the heat wave discussed in this study, 36% of seagrass meadows were damaged in Shark Bay, a world heritage listed area, and an estimated 2–9 Tg CO_2_ may have been released to the atmosphere^44^. Similarly, the 2003 heat wave in Europe resulted in significant disruptions to productivity across multiple forest ecosystems^45,46^, and the heat wave in 2012 in the NW Atlantic Ocean which led to marine species shifting their geographic distribution and seasonal cycles^23^. Additional flow-on effects include changes to key ecological processes, such as herbivory^14,35,42,47^, which maintained altered habitats, reduced resilience of tree populations to pests and pathogens^38^, altered forest structure^40,48^, and increased proximal predicted rate of wildfire spread^49^. As the frequency and spatial extent of heat waves continues to increase^1,50^, recovery times and persistence opportunities for many of these species may be further impacted.

Collectively, these results show a consistent, synchronous large spatial and taxonomic response throughout a terrestrial-marine ecoregion. We infer this from the spatial coherence of observations and consistency of findings across taxa, trophic groups, and ecosystems types within 1–2 seasons following the heat wave. Our results do not enable us to evaluate more detailed questions about the precise causality of responses to the heat wave, or the longer-term impacts or resilience. The rapid response of many taxa to heat stress indicate changes in abundance and condition are likely a direct consequence of the heat wave. However, for species with protracted change we are unable to determine if this is a direct response to the heat wave or an indirect response to changes in resource availability. Our results are consistent with some expected cross-taxa sensitivities, particularly for terrestrial woody plant species for which mortality increased with lifeforms progressing towards more mesic-affiliated taxa, from heathland shrubs (~2% mortality) to heathland trees (~18%) to woodland trees (~25%) to forest trees (~49%; Table 1). The coherence of marine and terrestrial responses to the heat wave also are suggestive that tree mortality patterns, while likely predisposed by drought, appear to have been triggered by the heat wave itself. Observations of changes in crown health corresponded with a prolonged heat wave in late February 2011^16^. These findings provide a foundation on which to build future experimental and observational studies regarding the specific nature of heat wave impacts, while simultaneously providing evidence for the potential of heat waves to trigger pervasive and spatially extensive biotic disruptions.

Understanding the coherence of the marine and terrestrial heat wave requires an appreciation of large-scale atmospheric and oceanic climatic drivers. Sea surface temperatures (SSTs) along the west coast of Australia are linked to the El Niño Southern Oscillation (ENSO), which influences the strength of southerly flowing currents via the Indonesian Flow Through. La Nina years are associated with a stronger Leeuwin current and warmer tropical SSTs at high latitude temperate reefs^51,52^. During the 2010–2011 Austral summer, an exceptionally strong La Nina event and northerly winds resulted in a surge in the Leeuwin current and abnormally high SSTs temperatures along the ocean margins of southwestern Australia^53^. In the terrestrial system, the warmer and dryer than average conditions in southwestern Australia leading up to the 2010–2011 event, follow a long-term warming and drying trend. While the warming trend has been largely attributed to increased anthropogenic greenhouse gas emissions^26,54^, the cause of the consistent reduction in rainfall since the 1970s is not as clear, with numerous studies identifying several factors as likely contributors. This includes natural variability^55^, changes in ocean temperatures^56,57^, land-use change^58^, a southern shift of storm tracks^59^, as well as snowfall increases in coastal east Antarctica^60^. The latest global climate projections^61^ and recent high resolution regional climate projections^62^ show a statistically significant decline in winter rainfall across southwestern Australia linked to few rain bearing fronts traversing the region, consistent with a southern shift of storm tracks^59^. However, compound terrestrial and marine heat waves in this region, and the degree to which they could be linked along a given terrestrial-marine continental boundary, remains uncertain. Nonetheless, the coherence of the marine and terrestrial patterns seen in the 2011 event is striking, and future evaluation of potential linkages is warranted.

The biotic disruptions that we document are notable in each of five aspects, in that our results: (1) build from a focus on single or co-dominant species studies^10,16–18^ to document responses across broad taxonomic lineages; (2) extend beyond physiological^1,11^ or phenological^1^ change to demographic disruptions; (3) move beyond focus on gradual ecological changes^12,22,30^ to specifically assess abrupt ecological change; (4) quantify changes from individual locations^16–18^ to spanning up to sub-continental scale; and (5) break down silos across historic disciplinary boundaries between marine^13,14,18,19,35,42,47^ and terrestrial^11,16^ ecology. That our results simultaneously show biotic responses in all five of these aspects provides evidence for the pervasive ecological vulnerability to a climate change-induced extreme event. We have documented and highlighted the breadth of taxa affected and quantified demographic change in abundance and mortality rates. Collectively, our results imply that the full extent of ecological vulnerability to projected heat waves is grossly underestimated.

## Methods

We evaluated the ecological responses associated with a subcontinental heat wave in 2010–2011 across an ecoregion of adjoining terrestrial and marine ecosystems along the Western Australian coast. To characterize physical heat wave conditions in both terrestrial and marine ecosystems, we compared 2011 temperature conditions with long-term averages (1971–2000). Impact on terrestrial and marine biota (N = 30 taxa) was then assessed via assimilating published reports and summarising using a meta-analytic framework.

*Climatic Data*. The daily maximum and minimum temperature and precipitation dataset used in this study is the Australian Bureau of Meteorology’s gridded observational product. The dataset has a resolution of 5 km and represents an interpolation from a network of weather stations across Australia, employing topography-resolved analysis methods to minimize uncertainty in fitting a surface to observations^63^. This dataset was chosen as it is the most reliable and widely used by numerous studies which focus on heat waves in Australia, e.g.^4,64–66^.

A terrestrial heat wave event within the climate record (1960–2014) was identified as three consecutive days during Austral summer months (November to March) where the maximum temperature was over the 90th percentile threshold (based on a 15-day analysis window). These calculations followed the ‘CTX90pct’ method^4^, a method well suited to capturing trends in heat waves in Australia. Heat wave days are calculated as the sum of all summer days that were identified in a heat wave event for a particular summer period (i.e. November 2010 to March 2011 was assigned as 2011 summer).

Sea Surface Temperature (SST) data are from Reynolds *et al*.^67^. This dataset combines *in-situ* observations and remotely sensed estimates to produce a SST dataset at a 1 by 1 degree resolution and is suitable for monitoring of weather and climate on a weekly time-scale since the 1990s. Whilst there are other blended *in-situ* and remotely sensed SSTs datasets such as that of Rayner *et al*.^68^, these are only available on a monthly time-scale, which was judged too coarse for capturing heat wave events. Therefore the dataset of Reynolds *et al*.^67^ was chosen because of its higher frequency and previous use in heat wave studies, e.g^5,69^.

*Biotic Data*. Our aim was to quantify pre- to post-heat wave demographic change in the abundance or mortality rates of individual taxa (see Extended Data Table 1 for detailed description of metrics). To quantify the magnitude of ecological impacts from the heat wave, data were sourced from peer-reviewed publications, government reports, and unpublished sources (Extended Data Table 1). Authors used the peer-reviewed literature, local knowledge, and professional contacts to identify data sources and develop a dataset of heat wave disruptions, encompassing 45 records of 30 terrestrial and marine taxa from within the impacted ecoregion. For marine data, a workshop was held shortly after the heat wave event^33^ and evidence of impacts, both quantitative and qualitative, were compiled. We relied on this report and participants’ subsequent publications for marine-based data. For terrestrial impacts, most data were unpublished and contributed by co-authors. To merit inclusion, data on heat wave impacts had to include information from October 2010 to July 2011 (start of the marine heat wave and end of the terrestrial heat wave responses), include an unimpacted contrast (typically prior measurement), and be from the impacted region of Western Australia (marine: latitudes 20–34 °S; terrestrial: 25–34 °S; Fig. 1, Fig S1). All such available data were included: taxa and data were not selected on the basis of an observed impact and were treated as a sample of taxa measured during the climate event. How the taxa examined were selected for survey in the first place varied for each study, typically species were keystone species, ecosystem dominants or threatened taxa. To be included, quantitative data had to report a pre to post comparison (10 studies) or a space for time substitution (1 study; Extended Data Table 1).

Data were extracted from studies recording taxon, location, mean, error, and sample size for pre and post heat wave measurements. Data were demographic in nature, including mortality rates of individual plants or changes in population abundance (cover of plants, mortality rates of individual plants, counts of animals; Extended Data Table 1). Where data included multiple pre-heat wave measurements we combined prior data or retained only the previous year (where time scales >2 years prior). Where studies included multiple taxa, spanned a major geographic divide (island groups) or distance (>100 km), we recorded each comparison separately (Extended Data Table 1). Taxa affected were categorized in relation to their ecosystem function (producers: photosynthetic organisms including algae, seagrass, coral, shrubs, and trees; consumers: non-photosynthetic organisms). We further divided organisms based on their pre-heat wave prevalence/abundance and life history attributes of distribution (tropical/temperate) and disturbance response (positive/negative). This resulted in taxa classified in terms of expected positive (increaser) or negative (decreaser) response to a heat wave. In all cases, we relativised data within contrasts to set the pre-heat wave levels to an equivalent point to facilitate comparisons.

Impact data varied among studies depending on the initial design of the survey and the nature of the taxon in question. All parameters had a direct bearing on population response, and included: survival, abundance, range change or fecundity. Biotic disruption records were aggregated by trophic levels (primary vs. secondary), producers or consumers where taxa were classified *a priori* expected to decrease in abundance following the heat wave from those expected to increase in abundance or distribution (range extension). We expressed all biotic disruptions as percent change post-event, and normalized all pre heat wave values to zero, thereby permitting assessment of heat wave impacts and comparison across taxa and disparate units of measurement (similar to other meta-analytic studies where the outcome measures from different experiments are standardised and put on the same scale^70^). Means and 95% confidence intervals by functional group were calculated using a mean weighted by the number of independent sample units in order to facilitate pre to post comparisons. Lack of confidence interval overlap was interpreted as strong evidence of a difference between groups, and confidence interval overlap but not including the mean was interpreted as moderate evidence^71^. The datasets generated during and/or analysed during the current study are available from the corresponding author on request.

## Electronic supplementary material

Supplementary Information

Dataset 1
