# Supplementary material for: Subcontinental heat wave triggers terrestrial and marine, multi-taxa responses
Source: Sci Rep. 2018 Aug 30;8:13094. doi: 10.1038/s41598-018-31236-5 (PMC6117366; doi:10.1038/s41598-018-31236-5)
Supplement: Supplementary file 1 — Supplementary Information [file 41598_2018_31236_MOESM1_ESM.docx]

**Supplementary information**

**Subcontinental heat wave triggers terrestrial and marine, multi-taxa responses**

Katinka X Ruthrof, David D Breshears, Joseph B Fontaine, Ray H Froend, George Matusick, Jatin Kala, Ben P Miller, Patrick J Mitchell, Shaun K Wilson, Mike van Keulen, Neal J Enright, Darin J Law, Thomas Wernberg and Giles E St J Hardy

Data management and analysis

*Standardised precipitation evapotranspiration index (SPEI) analysis.* Monthly climatic water deficits (1960-2014) were calculated using the standardised precipitation evapotranspiration index (SPEI)[^1^](#_ENREF_1). The SPEI is a simple climatic water balance based on the difference between precipitation and potential evapotranspiration (calculated from Thornthwaite’s equation), that is deseasonalised and subsequently standardised so that drought conditions at different locations, over a variety of pre-defined time scales, can be represented as probabilities and presented in a comparable fashion. By construction, the SPEI has a standard normal distribution, hence negative values represent periods of net negative water balance and the probability of exceeding values of -1 and -2 are approximately 0.16 and 0.02, respectively. In this analysis we present SPEI values computed over a 12-month time scale that represents the climatic water balance over the preceding 12 months for any given month.

**a**
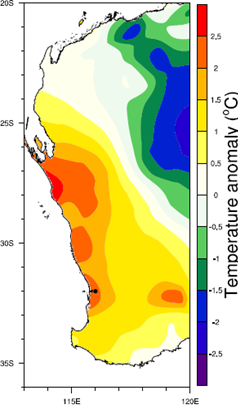
 **b**
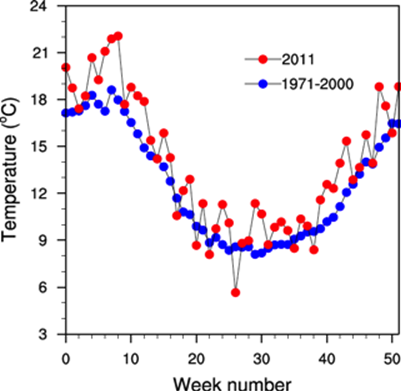


**Figure S1: Minimum temperature anomalies. a,** Spatial pattern of terrestrial minimum temperature anomaly for March 2011 relative to March 1971-2000 for the south west of Western Australia. **b,** Time series of weekly mean minimum temperature for 2011 (red dots) and 1971-2000 (blue dots) near Perth, Western Australia (32^o^S, 116^o^E) as shown by the black dot in panel, **a.** Figure was produced using the software NCAR Command Language (Version 6.4.0) [Software]. (2017). Boulder, Colorado: UCAR/NCAR/CISL/TDD. <http://dx.doi.org/10.5065/D6WD3XH5>

**a**


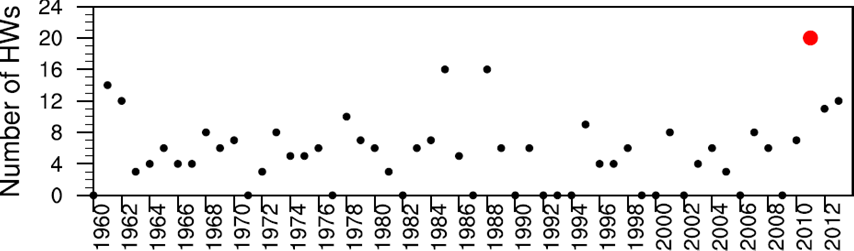


**b**


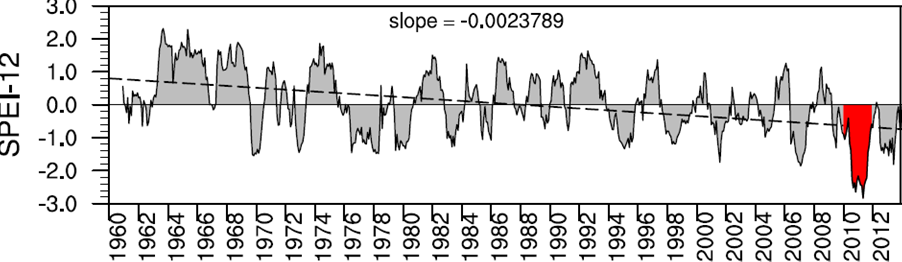


**Figure S2: Historical heat waves and standardized precipitation evapotranspiration index showing relative significance of event in 2011 (red) and trend over time. a,** The number of days experiencing heat wave conditions (heat wave days, see Heat wave Analysis above) from 1960 to 2013 during summer (November to March) computed near Perth, Western Australia (32^o^S, 116^o^E). The red dot shows the number of heat wave days in 2011. **b,** Standardized precipitation evapotranspiration index (SPEI) computed over twelve month timespans for each month from 1960-2012 near Perth, Western Australia (32^o^S, 116^o^E). The red shading shows the magnitude of SPEI during the heat wave in 2011.

**Extended Data Table 1 (separate file):** Full dataset for quantitative and qualitative studies on terrestrial and marine taxa affected by heat wave in 2011 in Western Australia. Table references [^2-12^](#_ENREF_2)**.**

References

1 Vicente-Serrano, S. M., Lasanta, T. & Gracia, C. Aridification determines changes in forest growth in *Pinus halepensis* forests under semiarid Mediterranean climate conditions. *Agric. For. Meteorol.* **150**, 614-628, doi:10.1016/j.agrformet.2010.02.002 (2010).

2 Cannell, B. L., Chambers, L. E., Wooller, R. D. & Bradley, J. S. Poorer breeding by little penguins near Perth, Western Australia is correlated with above average sea surface temperatures and a stronger Leeuwin Current. *Mar. Freshw. Res.* **63**, 914-925, doi:10.1071/mf12139 (2012).

3 Depczynski, M. *et al.* Bleaching, coral mortality and subsequent survivorship on a West Australian fringing reef. *Coral Reefs* **32**, 233-238, doi:10.1007/s00338-012-0974-0 (2013).

4 Byrne, M., Barrett, G., Blythman, M., Finn, H. & Williams, M. Cocky Count: a community-based survey for Carnaby’s Black-Cockatoo (*Calyptorhynchus latirostris*) and Forest Red-tailed Black-Cockatoo (*Calyptorhynchus banksii naso*). (BirdLife Australia, Floreat, Western Australia, 2015).

5 Matusick, G., Ruthrof, K. X., Brouwers, N. C., Dell, B. & Hardy, G. S. Sudden forest canopy collapse corresponding with extreme drought and heat in a mediterranean-type eucalypt forest in southwestern Australia. *European Journal of Forest Research* **132**, 497-510, doi:10.1007/s10342-013-0690-5 (2013).

6 Moore, J. A. Y. *et al.* Unprecedented Mass Bleaching and Loss of Coral across 12 degrees of Latitude in Western Australia in 2010-11. *PLoS One* **7**, 11, doi:10.1371/journal.pone.0051807 (2012).

7 Seaton, S., Matusick, G., Ruthrof, K. X. & Hardy, G. Outbreak of *Phoracantha semipunctata* in response to severe drought in a mediterranean eucalyptus forest. *Forests* **6**, 3868-3881, doi:10.3390/f6113868 (2015).

8 Smale, D. A. & Wernberg, T. Extreme climatic event drives range contraction of a habitat-forming species. *Proc. R. Soc. B-Biol. Sci.* **280**, 9, doi:10.1098/rspb.2012.2829 (2013).

9 Thomson, J. A. *et al.* Extreme temperatures, foundation species, and abrupt ecosystem change: an example from an iconic seagrass ecosystem. *Glob. Change Biol.* **21**, 1463-1474, doi:10.1111/gcb.12694 (2015).

10 Wernberg, T. *et al.* An extreme climatic event alters marine ecosystem structure in a global biodiversity hotspot. *Nat. Clim. Chang.* **3**, 78-82, doi:10.1038/nclimate1627 (2013).

11 Pearce, A. *et al.* The “marine heat wave” off Western Australia during the summer of 2010/11. 40 (Fisheries Research Division Western Australian Fisheries and Marine Research Laboratories, North Beach, Western Australia, 2011).

12 Challis, A., Stevens, J. C., McGrath, G. & Miller, B. P. Plant and environmental factors associated with drought-induced mortality in two facultative phreatophytic trees. *Plant Soil* **404**, 157-172, doi:10.1007/s11104-016-2793-5 (2016).
